# Transient Loss of Protection Afforded by a Live Attenuated Non-typhoidal *Salmonella Vaccine* in Mice Co-infected with Malaria

**DOI:** 10.1371/journal.pntd.0004027

**Published:** 2015-09-14

**Authors:** Jason P. Mooney, Seung-Joo Lee, Kristen L. Lokken, Minelva R. Nanton, Sean-Paul Nuccio, Stephen J. McSorley, Renée M. Tsolis

**Affiliations:** 1 Department of Microbiology & Immunology, School of Medicine, University of California Davis, Davis, California, United States of America; 2 Center for Comparative Medicine, Department of Anatomy, Physiology and Cell Biology, School of Veterinary Medicine, University of California Davis, Davis, California, United States of America; Massachusetts General Hospital, UNITED STATES

## Abstract

In immunocompetent individuals, non-typhoidal *Salmonella* serovars (NTS) are associated with gastroenteritis, however, there is currently an epidemic of NTS bloodstream infections in sub-Saharan Africa. *Plasmodium falciparum* malaria is an important risk factor for invasive NTS bloodstream in African children. Here we investigated whether a live, attenuated *Salmonella* vaccine could be protective in mice, in the setting of concurrent malaria. Surprisingly, mice acutely infected with the nonlethal malaria parasite *Plasmodium yoelii* 17XNL exhibited a profound loss of protective immunity to NTS, but vaccine-mediated protection was restored after resolution of malaria. Absence of protective immunity during acute malaria correlated with maintenance of antibodies to NTS, but a marked reduction in effector capability of *Salmonella*-specific CD4 and CD8 T cells. Further, increased expression of the inhibitory molecule PD1 was identified on memory CD4 T cells induced by vaccination. Blockade of IL-10 restored protection against *S*. Typhimurium, without restoring CD4 T cell effector function. Simultaneous blockade of CTLA-4, LAG3, and PDL1 restored IFN-γ production by vaccine-induced memory CD4 T cells but was not sufficient to restore protection. Together, these data demonstrate that malaria parasite infection induces a temporary loss of an established adaptive immune response via multiple mechanisms, and suggest that in the setting of acute malaria, protection against NTS mediated by live vaccines may be interrupted.

## Author Summary

In children, malaria is a predisposing factor for invasive bacterial infections with non-typhoidal *Salmonella* (NTS) serovars, a frequent cause of morbidity and mortality in sub-Saharan Africa. Since development of vaccines against NTS has been proposed as a strategy to protect African children against disseminated NTS infection, we interrogated the effect of malaria on vaccine-induced memory responses to NTS. Our results from a mouse infection model show that infection with malaria parasites temporarily suspends protective immunity conferred by a live, attenuated vaccine and suppresses adaptive immune responses to NTS that are mediated by T cells. These results suggest that in the setting of acute malaria, live attenuated NTS vaccines may lose their effectiveness.

## Introduction

In immunocompetent individuals, non-typhoidal *Salmonella* serovars (NTS) cause gastroenteritis, a localized enteric infection characterized by intestinal neutrophil recruitment and diarrhea [1]. NTS gastroenteritis is the single most common cause of death from diarrheal disease associated with viruses, parasites or bacteria in the US [2] and high profile outbreaks provide a good visibility of this public health problem. Recently it has become more widely recognized that NTS infections have an enormous impact in developing countries, particularly in Sub-Saharan Africa. NTS are an important cause of gastroenteritis in Sub-Saharan Africa [3]. However, in addition these pathogens are often the most common cause of bloodstream infections, with *Salmonella enterica* serovars Enteritidis and Typhimurium (*S*. Enteritidis and *S*. Typhimurium) accounting for the majority of cases [4–8]. This syndrome, known as NTS bacteremia, is not a diarrheal disease, as symptoms of gastroenteritis are commonly absent [9]. Development of NTS bacteremia in African children is clinically associated with young age as well as with underlying conditions, including *Plasmodium falciparum* malaria, malnutrition, acquired immunodeficiency syndrome (AIDS) and anemia [9]. Of particular concern for treatment is the prevalence in this region of a novel genotype of *S*. Typhimurium, ST313, that is resistant to multiple antibiotics [10,11].

Currently there is strong interest in development of vaccines to prevent disseminated NTS infection, and both glycoconjugates and live, attenuated vaccines are currently in preclinical testing [9,12]. However, it is poorly understood how comorbidities such as malaria might impact protection afforded by such vaccines. To address this question, we used murine models to investigate whether existing immunity against disseminated infection conferred by a live, attenuated *S*. Typhimurium vaccine remains effective in the setting of malaria parasite infection.

## Materials and Methods

### Ethics statement

Experiments with mice were carried out in strict accordance with the recommendations in the Guide for Care and Use of Laboratory Animals of the National Institute of Health and were approved by the Institutional Animal Care and Use Committees at the University of California at Davis under protocols 16597, 16612, 16932 and 18183.

### Mouse strains

Specific pathogen free (SPF) 6–8 week-old female C57BL/6J and CBA/J mice were purchased from the Jackson Laboratory (Bar Harbor, Maine) or C57BL/6NCr were purchased from the National Cancer Institute (Frederick, MD). Mice were housed under SPF conditions by the UC Davis Center for Laboratory Animal Science; receiving irradiated rodent chow and sterile drinking water *ad libitum*. C57BL/6J-*Slc11a1* ^+/+^ (also known as *Nramp1*) mice were obtained from Greg Barton at the University of California Berkeley, rederived by the UC Davis Mouse Biology Program and backcrossed to C57BL/6J under barrier conditions by the UC Davis Institute for Regenerative Cures. C57BL/6J-*Slc11a1* ^+/+^ genotypes from tail biopsies were determined using real time PCR with specific probes designed for the gene *Slc11a1* (Transnetyx, Cordova, TN).

### *Plasmodium yoelii* 17XNL

*(P*. *yoelii)* Parasites were kindly provided by Ana Rodriguez and Shirley Luckhart. Parasite stocks were made by passage in CD-1 mice, and harvested when mice had 5–10% parasitemia. For co-infection experiments, mice were inoculated i.p. on day 0 with approximately 4x10^7^ infected red blood cells (iRBCs) in 0.1 ml of saline. Mock-infected controls were injected i.p. with an equivalent amount of blood from uninfected CD-1 mice.

### Bacterial strains

*Salmonella enterica* serovar Typhimurium (*S*. Typhimurium) BRD509 strain [13] was kindly provided by Dr. D. Xu, University of Glasgow, Glasgow, U.K. To generate the *S*. Typhimurium BRD509 strain expressing the 2W1S epitope (EAWGALANWAVDSA) (strain SPN555), P22 generalized transduction [14,15] was performed using as a donor an *S*. Typhimurium SL1344 strain expressing 2W1S [16], which was a kind gift from Dr. M. Jenkins, University of Minnesota, Minneapolis, MN. *Salmonella* were cultured overnight in Luria-Bertani (LB) broth (Difco, BD Diagnostics, Sparks, MD) and diluted in PBS after estimation of bacterial concentration using a spectrophotometer. For vaccination, 5x10^5^ colony-forming units (CFU) of BRD509 was administered i.v.. For tetramer-tracking experiments, C57BL/6 mice were vaccinated with 5x10^5^ CFU of BRD509-2W1S. For challenge, virulent *S*. Typhimurium strain SL1344 *phoN*::Kan^R^ (strain SW1096, provided by Dr. S. Winter, University of Texas Southwestern Medical Center, Dallas TX) was administered in malaria-infected or control mice i.v. using 750–1000 CFU or administered in CBA/J mice via oral gavage using 1x10^8^ CFU one day after oral administration of 20mg streptomycin (Sigma) in 0.1mL water. Inoculates were cultured for 16 h aerobically at 37°C. In all experiments, the final bacterial dose administered was confirmed by plating onto LB agar plates.

### Microbial readouts of infection

Parasitemia was determined by counting the percentage of *P*. *yoelii* iRBCs on thin blood smears stained with Giemsa (Acros Organics, NJ). Whole blood was collected with heparinized syringes and complete blood counts were analyzed by the UC Davis Comparative Pathology Laboratory using the Drew Scientific 950 FS Hematological Analyzer. To determine the numbers of viable *Salmonella*, livers and spleens were homogenized in PBS using an Ultra Turrax T25 Basic mixer (IKA). Blood was collected from hearts with a heparinized needle, plasma was removed and incubated for 10min with 120μL of 1% Triton X-100 in PBS at room temperature. Homogenates were serially diluted and plated on LB agar plates containing 100mg/L kanamycin. After overnight growth at 37°C, CFU/gram of tissue was calculated.

### Cell processing

Spleens, mesenteric lymph nodes (MesLN), and peripheral lymph nodes (PLNs, inguinal, axillary, brachial) were crushed through nylon mesh. Red blood cells (RBCs) in spleen were lysed with ACK lysing buffer (Lonza, Walkersville, MD), and then washed with 2% FBS/PBS. Livers were perfused, crushed through a cell strainer (BD Biosciences, San Diego, CA), and resuspended in 35% Percoll (Sigma-Aldrich, St. Louis, MO). After centrifugation, pelleted cells were treated with ACK lysing buffer, and then washed with 2% FBS/PBS.

### Tracking 2W1S-specific endogenous CD4 T cell response using class-II tetramer and analyzing cytokine production by specific CD4 T cells

Tracking *Salmonella* specific CD4 T cell response using MHC class II tetramers was performed as described previously [17]. Malaria-infected or control mice were injected i.v. with lysates of 10^8^ CFU heat-killed *Salmonella*. Four hours later, spleens and livers were collected and processed into single cell suspensions. Cells were stained with PE-conjugated 2W1S::I-A^b^ tetramer in the presence of Fc block (culture supernatant from the 2.4G2 hybridoma, 2% mouse serum, 2% rat serum) room temperature for one hour. After washing with 2% FBS/PBS, cells were strained with fluorochrome-conjugated antibodies specific for CD3, CD4, CD8, and CD44 (eBioscience, San Diego, CA). For intracellular cytokine staining, cells were surface stained, and then treated with Foxp3/Transcription Staining buffer set (eBioscience) according to the manufacturer’s recommendation. Permeabilized cells were stained with fluorochrome-conjugated antibodies specific for IFN-γ (eBioscience). Cells were then analyzed by flow cytometry using a BD LSR Fortessa (BD Biosciences). All data sets were analyzed using FlowJo software (Tree star, San Carlos, CA).

### Examining *Salmonella*-specific T cell response *ex vivo*

MultiscreenHTS ELISPOT plates (Millipore, Billerica, MA) were coated with purified anti-mouse IFN-γ (BD Biosciences). Total CD4 T cells from malaria-infected or control splenocytes were stained with PE-conjugated anti-mouse CD4 (eBioscience) and then purified using the EasySep Mouse PE Positive Selection kit (STEMCELL Technologies, Vancouver, Canada) according to the manufacturer’s protocol. Total CD8 T cells were isolated from unbound fraction of CD4-PE positive selection using the EasySep Mouse CD8 Enrichment kit (STEMCELL Technologies) according to the manufacturer’s guidance. The purity of CD4 T cells and CD8 T cells was measured by flow cytometry and was typically >80%. Enriched CD4 or CD8 T cells were added to plates in the presence of 8x10^5^ irradiated naïve splenocytes. Cells were restimulated with 10μM peptides (**Table 1**) at 37°C overnight. For CD8 T cell peptides, epitopes in Sse proteins were discovered by generating T cell lines and scanning for reactivity with overlapping peptides as described previously [18]. Bound IFN-γ was detected by biotin-conjugated anti-mouse IFN-γ (BD Biosciences), followed by AP-conjugated streptavidin (BD Biosciences). Bound IFN-γ antibodies were visualized using One-step NBT-BCIP substrate (Thermo, Rockford, IL) and plates were analyzed using an AID iSpot Reader Spectrum (AID US, San Diego, CA). The number of IFN-γ producing cells was normalized to the total number of enriched CD4 or CD8 T cells as determined by flow cytometry.

**Table 1:** Peptides used in this study.

	Peptide	Sequence	Source
CD4	Flagellin 427–441	VQNRFNSAITNLGNT	McSorley SJ 2000 [19]
	SseI 268–280	LIYYTDFSNSSIA	Lee SJ 2012 [18,19]
	SseJ 329–341	CYYETADAFKVIM	Lee SJ 2012 [18,19]
	2W1S	EAWGALANWAVDSA	Rees W 1999 [20]
CD8	SseI 68–75	VVSRFELL	This study
	SseJ 273–281	GGVNNVLVM	This study
	SseB 54–62	QAIANNKFI	This study

### *Salmonella*-specific antibody responses

Blood was collected retro-orbitally from malaria-infected or control mice. Sera were prepared by centrifugation. High-protein binding plates (Corning, Tewksbury, MA) were coated overnight with heat-killed *Salmonella* diluted in 0.1M NaHCO_3_. After incubation in 10% FBS/PBS for one hour at 37°C, the plates were washed twice with PBS/0.05% Tween 20, and serum samples were added in serial dilutions in 10% FBS/PBS. After incubation for two hours at 37°C, the plates were washed four times before the addition of biotin-conjugated anti-mouse IgG2c antibody (BD Bioscience). After incubation for one hour at 37°C, the plates were washed six times and incubated for one hour at 37°C with HRP-conjugated streptavidin (Sigma-Aldrich). The plates were then washed eight times and an HRP substrate (*O*-phenylenediamine dihydrochloride, Sigma-Aldrich) was used to develop the plates. After sufficient color-change was observed, the reaction was stopped by adding 100μL 1M H_2_SO_4_. The plates were analyzed using a spectrophotometer (SpectraMax M2, Molecular Devices, Sunnyvale, CA).

### Antibody blockade of PDL-1/LAG3/CTLA-4 or IL-10

For the PDL-1/LAG3/CTLA-4-blocking experiment with *in vivo* stimulation at day 14, malaria-infected and control mice were treated i.p. with a 300μL cocktail containing 400 μg of anti-mouse PDL-1, 400 μg of anti-mouse LAG3 and 400 μg of anti-mouse CTLA-4, or isotype control antibodies (BioXcell, West Lebanon, NH) 7, 9, 11, and 13 days after malaria infection. For the experiment in which mice were challenged at day 14 after malaria infection, an additional injection of 260μg of antibody was given at day 15. For the IL-10 blocking experiment, a total of 600 μg of rat anti-mouse IL-10 immunoglobulin G1 (IgG1) kappa (eBioscience, San Diego, CA) or isotype control was given i.p. in the following manner; 300 μg on day 13, 200 μg on day 14, and 100 μg on day 15.

### RNA extraction, reverse transcription-PCR (RT-PCR), and real-time PCR

Animal tissues were frozen in liquid nitrogen at necropsy and stored at -80°C. RNA was extracted from tissue as described previously [21] using Tri-Reagent (Molecular Research Center) according to the instructions of the manufacturer. All RNA was treated with DNAseI (Ambion) to remove genomic DNA contamination. For a quantitative analysis of mRNA levels, 1μg of total RNA from each sample was reverse transcribed in a 50-μL volume, using reagents from Applied Biosystems, and 4 μl of cDNA was used for each real-time reaction. RT-PCR was performed using a ViiA 7 Real-Time PCR System (Applied Biosystems) and amplicons were detected via intercalation of SYBR green dye (Applied Biosystems). Data was analyzed by using the comparative threshold cycle (C_T_) method (Applied Biosystems). Target gene transcription of each sample was normalized to the respective levels of β-Actin mRNA and represented as fold change (2^-ΔΔCT^) over gene expression in control animals. Primers used in this study were the following: β-Actin forward (AGAGGGAAATCGTGCGTGAC) β-Actin reverse (CAATAGTGATGACCTGGCCGT), IFNγ forward (CAACAGCAAGGCGAAAAAGGATGC) IFNγ reverse (CCCCGAATCAGCAGCGACTCC), Il10 forward (GGTTGCCAAGCCTTATCGGA) Il10 reverse (ACCTGCTCCACTGCCTTGCT).

### Statistical analysis

The statistical significance of differences between groups was determined by a Student's *t* test on logarithmically transformed data. For data sets in which all values were the same (zero), statistical significance was determined by a Mann-Whitney U test. A *P* value of 0.05 or less was considered to be significant. All data were analyzed using two-tailed tests.

## Results

### Transient loss in vaccine-mediated protection against *Salmonella* during acute malaria

In sub-Saharan Africa, current or recent malaria are associated with increased risk for invasive bacterial infections, including those with NTS [22]. While previous studies have shown that clinical malaria may impair development of protective immunity after vaccination [23], few studies have addressed whether acute malaria impacts existing acquired immunity. Therefore, to examine how current or recent malaria would effect existing vaccine mediated protection against *S*. Typhimurium, we made use of a mouse model in which protective immunity is induced in the absence of malaria, via administration of a well-characterized live attenuated NTS vaccine (strain BRD509, *ΔaroA ΔaroD*) [24–28]. We then interrogated the effect of malaria on established immunity to *S*. Typhimurium.

**Fig 1:**
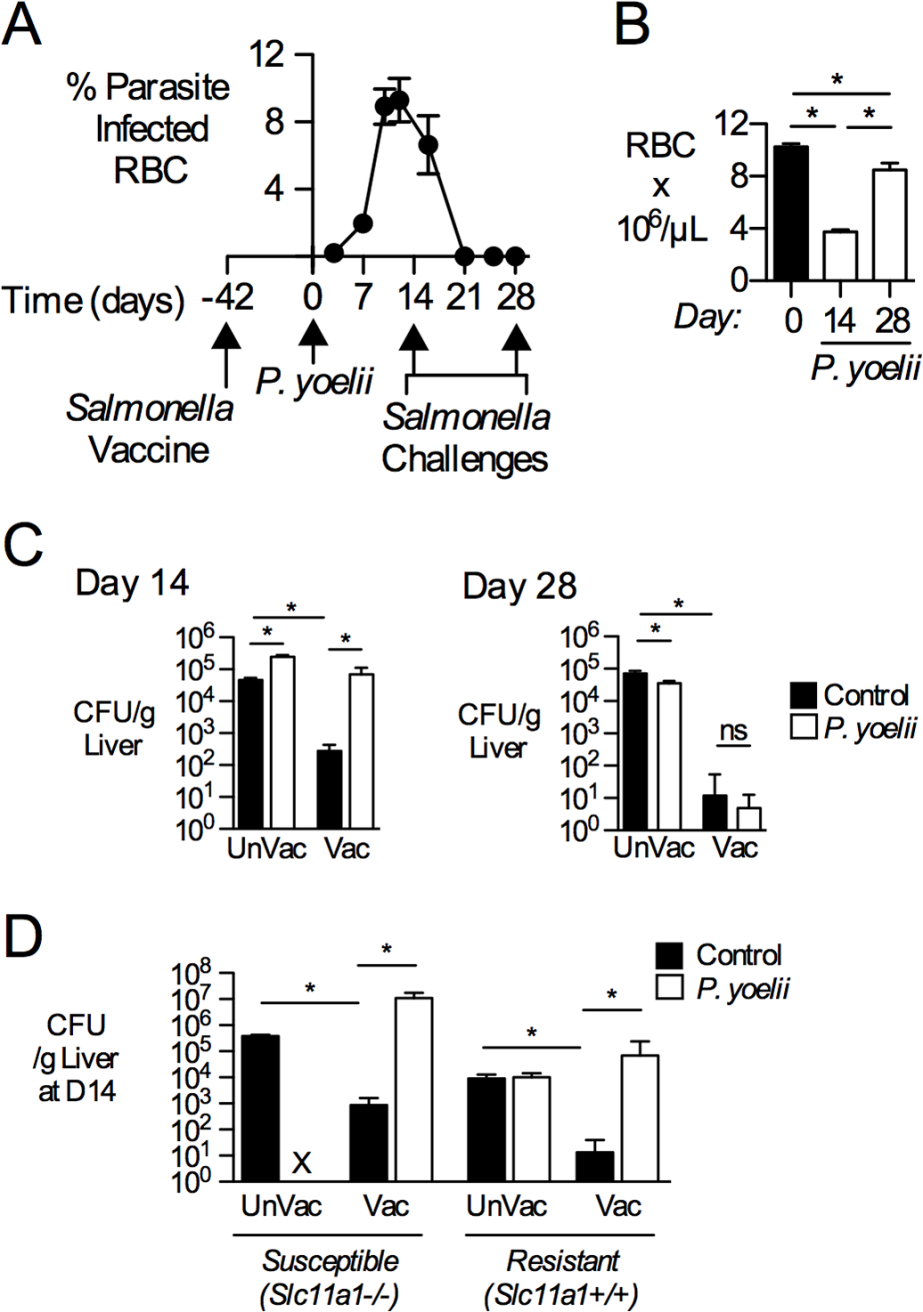
Transient loss in protection to *Salmonella* challenge during peak malaria-parasite infection in *Salmonella*-vaccinated mice. C57BL/6 mice were vaccinated i.v. with 5x10^5^ CFU attenuated *S*. Typhimurium BRD509 (Vac). Forty-two days later, a group of vaccinated mice was inoculated i.p. with blood containing 4x10^7^ *P*. *yoelii*-infected red blood cells (RBC) and the rest were given control blood. (**A**) Parasite burden, shown as % infected red blood cells (RBCs) (n = 25). Data represent Mean±SEM. (**B**) RBC concentrations (10^6^/uL) at either 14 or 28 days after malaria parasite inoculation as determined by complete blood counts (n = 4–8). Bars represent the Mean+SEM. (**C**) Mice were challenged with 750–1000 CFU of virulent *S*. Typhimurium via the i.v. route at either 14 days (**C**, left) or 28 days (**C**, right) after *P*. *yoelii* infection. At 3 d post-challenge, bacterial burden was determined in livers from mice infected with *S*. Typhimurium only (Control) or co-infected (*P*. *yoelii*). (**D**) C57BL/6 (n = 5), which are *Nramp1*/*Slc11a1* ^*-/-*^, or C57BL/6 expressing a functional allele of *Slc11a1* (n = 3–13), were vaccinated, then inoculated with *P*. *yoelii* and challenged i.v. with virulent *S*. Typhimurium at 14 days after *P*. *yoelii* infection. *S*. Typhimurium burden was quantified in the liver at 3 days after challenge with virulent *S*. Typhimurium. X, not performed. Data represent Mean+SEM (n = 4–8). Significance of differences between groups was determined using a Student’s *t* test (*, *p*<0.05; ns, not significant).

To interrogate effects of malaria on vaccine induced immunity to *S*. Typhimurium, C57BL/6 mice were immunized via the intravenous (i.v.) route with strain BRD509, as this route induces Th1-dependent immunity in the mouse [27]. After 6 weeks, vaccinated mice and sham-treated controls were inoculated with the non-lethal murine malaria parasite *Plasmodium yoelii* subspecies *yoelii* 17XNL (*P*. *yoelii*) by intraperitoneal (i.p.) injection of parasite-infected erythrocytes or an equal volume of uninfected murine red blood cells. Peak parasite burden, as determined by microscopy of thin blood smears, occurred at 12 days post *P*. *yoelii* inoculation, and resolution below the limit of detection occurred at 21 days (**Fig 1A**). *P*. *yoelii*-infected mice at 14 days post infection exhibited characteristic responses to malaria parasite infection including splenomegaly, anemia, and an increase in circulating lymphocytes (**S1 Fig**). These changes resolved by day 28, (**Figs 1B and S1**), when infectious blood-stage parasites could no longer be detected via transfer of whole blood to naïve mice (**S1C Fig**). Therefore, in subsequent experiments, we challenged *S*. Typhimurium-vaccinated mice around peak parasite infection, at day 14, and after the resolution of parasite infection, at day 28. Vaccinated or unvaccinated control mice were inoculated i.v. with 750–1000 colony forming units (CFU) of virulent SL1344 containing a selectable marker (SL1344 *phoN*::Kan^R^). Unvaccinated mice that were coinfected with *S*. Typhimurium and *P*. *yoelii* exhibited marked morbidity after 3 days post *S*. Typhimurium challenge (data not shown) and therefore experiments were terminated at this time. In vaccinated mice challenged with *S*. Typhimurium in the absence of parasite infection, bacterial colonization of the liver decreased by nearly two orders of magnitude compared to unvaccinated controls **(Fig 1C)**, and splenic colonization decreased by three orders of magnitude (**S2A Fig**). In contrast, vaccinated mice challenged with *S*. Typhimurium at day 14 of malaria parasite infection had significantly reduced protection, showing a 264-fold increase in bacterial burden in the liver compared to controls not infected with *P*. *yoelii* **(Fig 1C)** and a 31-fold increase in the spleen **(S2A Fig)**. Interestingly, vaccine-mediated protection was restored in both liver **(Fig 1C)** and spleen **(S2B Fig)** when mice were challenged with *S*. Typhimurium after resolution of malaria parasite infection (at day 28 after malaria parasite infection). Loss of vaccine-mediated protection during malaria parasite infection was independent of *Slc11a1* (*Nramp1*) genotype and route of *S*. Typhimurium infection, as we observed the same effect in both C57BL/6 *Slc11a1* ^*+/+*^ mice with i.v. infection routes and in CBA mice with intragastric (i.g.) routes (**Figs 1D, S2C** and **S3**). Notably, while vaccination protected both *Slc11a1* ^*+/+*^ and *Slc11a1* ^*-/-*^ mice from bacteremia, this protection was lost in *P*. *yoelii*-infected mice (**Fig 1D**). Together, these data show that protection conferred by a live, attenuated *S*. Typhimurium vaccine is lost during acute malaria parasite infection.

### Levels of circulating antibodies are unchanged during malaria

Vaccination against *S*. Typhimurium elicits a robust B cell response, which is important for control of secondary infections [29]. The generation of *S*. Typhimurium-specific antibodies involves T cell-dependent isotype class switching to IgG2c [30]. Given the importance of this switched antibody response in controlling the spread of secondary infection, we determined the effect of malaria parasite infection on vaccine-induced anti-*Salmonella* IgG2c titers. Circulating levels of IgG2c at 14 and 28 days after *P*. *yoelii* infection did not differ from non-infected mice **(S4 Fig)**, suggesting that the observed loss of protection does not result from depletion of these *Salmonella*-specific antibodies during acute malaria. Consistent with presence of class-switched IgG2c, levels of *Salmonella*-specific IgM were low and were not strongly affected by *P*. *yoelii* infection (**S4 Fig**).

### IFN-γ production by *Salmonella*-specific T cells is reduced during peak malaria

**Fig 2:**
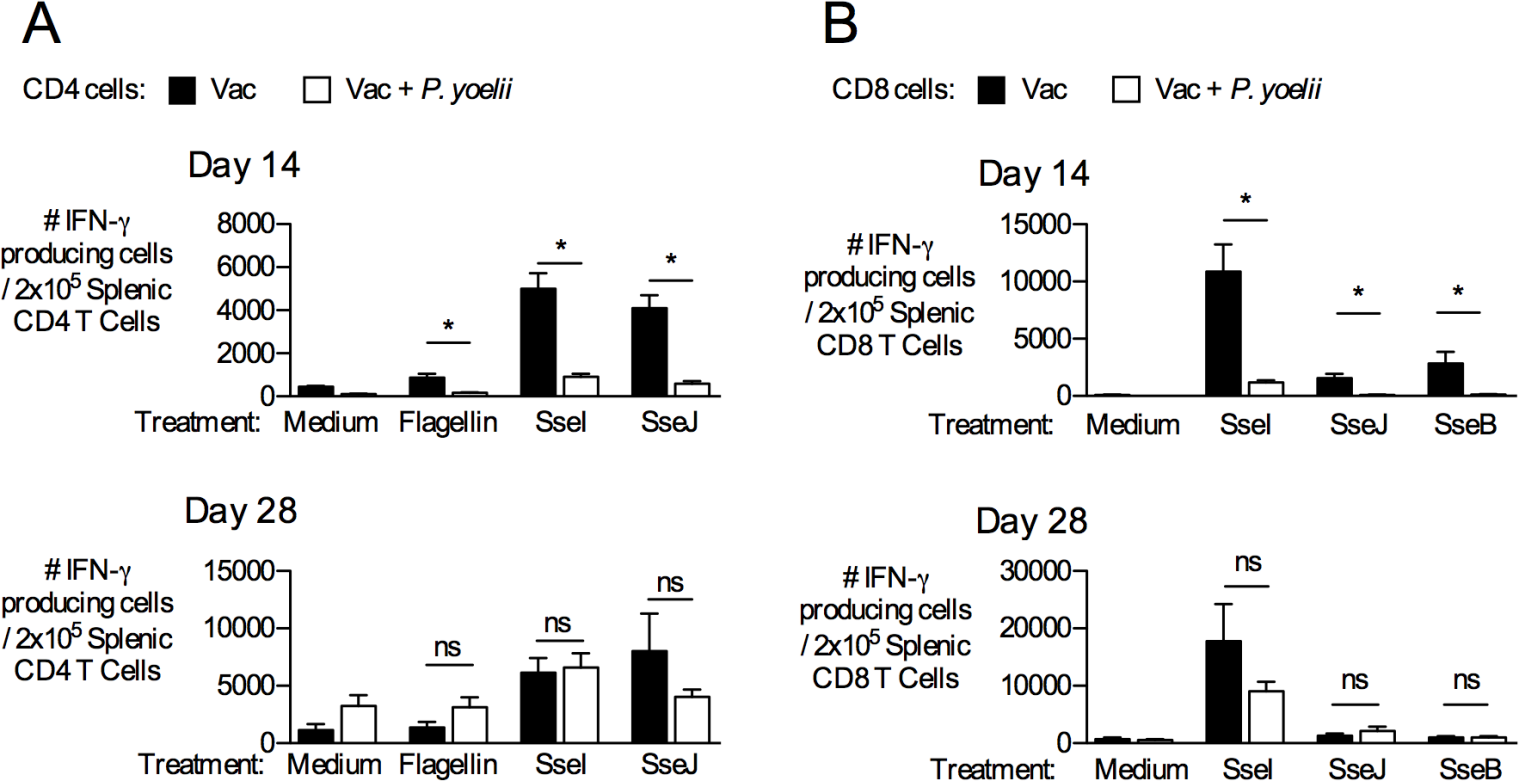
IFN-γ-producing capability of *Salmonella*-specific CD4 and CD8 T cells is reduced during peak malaria-parasite infection upon *ex vivo* stimulation. C57BL/6 mice were vaccinated with *S*. Typhimurium BRD509 (Vac). On day 42, these mice were inoculated with *P*. *yoelii* as described above. (**A**) At either 14 d (top) or 28 d post-malaria parasite infection (bottom), CD4 T cells were enriched from spleens of *P*. *yoelii*-infected or control mice. CD4 T cells were restimulated with Flagellin, SseI, or SseJ peptides (Table 1) in the presence of irradiated naïve splenocytes, and IFN-γ production was examined. Anti-CD3/anti-CD28 antibodies and no peptide (medium) wells constituted the positive and negative controls, respectively. Bar graphs show the number of IFN-γ-producing CD4 T cells per 2x10^5^ CD4 T cells. (**B**) CD8 T cells were enriched from the spleens of the same mice, and stimulated with peptides corresponding to Class I epitopes from SseB, SseI and SseJ (Table 1). Data represent Mean+SEM of three independent experiments (n = 9). Significance of differences between groups was determined using a Student’s *t* test (*, *p*<0.05; ns, not significant).

Adaptive immunity to *Salmonella* involves both CD4 and CD8 T cells and largely depends upon the production of IFN-γ [31]. Therefore, we determined the effect of active malaria infection on the function of memory T cells specific for a variety of natural *S*. Typhimurium MHC Class II epitopes from the target antigens flagellin, SseI and SseJ. Splenic CD4 T cells were enriched by immunomagnetic separation from BRD509-vaccinated mice that were infected with *P*. *yoelii* or mock treated and then stimulated *ex vivo* with exogenous *S*. Typhimurium epitopes (Table 1) in an IFN-γ-coated ELISPOT plate. In order to enumerate the number of IFN-γ producing CD4 T cells per well, we used flow cytometry **(Fig 2)**. After stimulation, we observed a marked decrease in the number of IFN-γ-producing CD4 T cells responding to flagellin, SseI, and SseJ (Table 1) at day 14 but these responses had largely recovered after the resolution of parasite infection (day 28) **(Fig 2A)**. *P*. *yoelii* infection alone did not affect numbers of IFN-γ-producing CD4 T cells in non-stimulated control samples (medium) **(Fig 2A).** *P*. *yoelii* infection had a similar suppressive effect on the function of splenic CD8 T cells responding to newly-identified Class I epitopes of SseI, SseJ and SseB **(Table 1 and Fig 2B),** that was restored upon resolution of parasitemia at day 28. Together, these data show that malaria parasite infection temporarily inhibits the ability of *Salmonella*-specific CD4 and CD8 T cells to produce IFN-γ upon re-encountering antigen.

**Fig 3:**
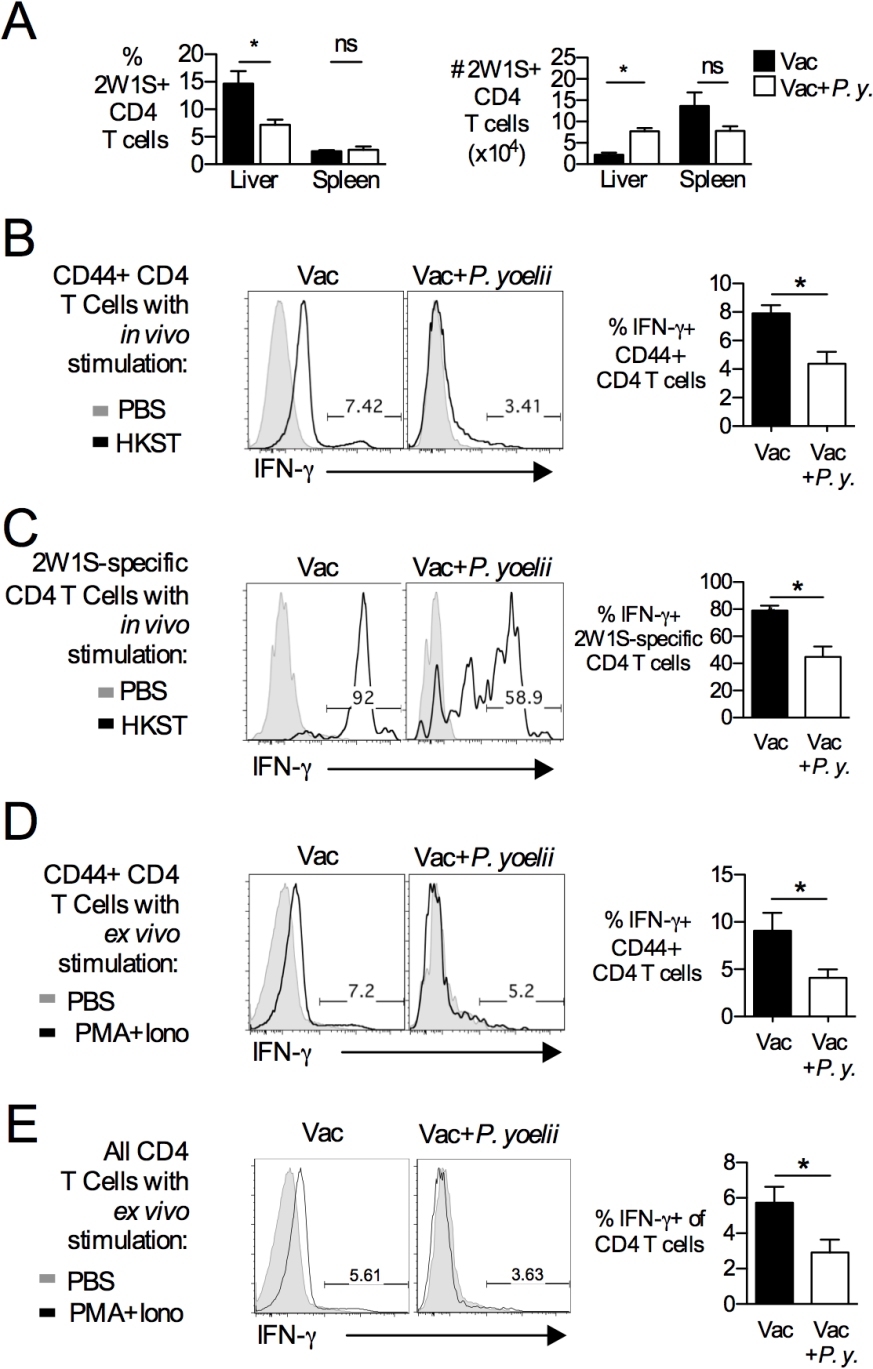
IFN-γ-producing capability of *Salmonella*-specific CD4 T cells is reduced during peak malaria-parasite infection. Mice were vaccinated with *S*. Typhimurium BRD509::2W1S (Vac). On day 42, vaccinated mice were infected with *P*. *yoelii* (*P*.*y*.). At 14 days post-malaria parasite infection, cells were analyzed. (**A**) Spleens and livers were analyzed by flow cytometry. Using 2W1S::I-A^b^ tetramer staining on CD4 T cells, we evaluated both the percentage (%) and absolute number (#) of 2W1S-specific CD4 T cell populations in the liver and spleen. Data are shown as Mean+SEM (n = 3–4). Representative dot plots are shown in S5 Fig. To simulate CD4 T cells *in vivo*, *P*. *yoelii*-infected or control mice were treated i.v. with heat-killed *S*. Typhimurium lysates (+ HKST) or PBS. After 4 hours, splenic CD44+ CD4 T cells (**B**) and 2W1S-specific CD4 T cells (**C**) were analyzed by flow cytometry for IFN-γ production. Data represent Mean+SEM of three independent experiments (n = 9). To assess antigen-independent stimulation, splenic CD4 T cells were stimulated with PMA and Ionomycin (Iono) for 4 hours. IFN-γ production was evaluated by flow cytometry for antigen-experienced (CD44+) **(D)** or all CD4 T cells **(E)**. Bars represent Mean+SEM (n = 3–4). Significance of differences between groups was determined using a Student’s *t* test (*, *p*<0.05; ns, not significant).

Based on the more prominent role of CD4 T cells in adaptive immunity to *S*. Typhimurium in mouse models [32,33], we focused our attention on suppression of CD4 T cell memory during acute malaria. In order to directly enumerate antigen-specific CD4 T cells *in vivo*, mice were initially vaccinated with a live vaccine strain of *S*. Typhimurium expressing the MHC Class II epitope 2W1S (BRD509::2W1S, strain SPN555) and subsequently infected with *P*. *yoelii*. Using this approach, the effect of parasite infection on the frequency and function of 2W1S-specific CD4 T cells was determined using a 2W1S MHC class-II tetramer [34]. While the frequency of tetramer-positive CD4 T cells decreased in the liver, the absolute number of 2W1S-specific cells was actually higher during malaria infection **(Fig 3A).** This alteration was limited to the liver, since both the total number and frequency of 2W1S-specific CD4 T cells in spleen and lymph nodes (mesenteric and peripheral LN) was unchanged during active malaria infection **(Figs 3A and S5).** The reduced proportion (%) of 2W1S+ cells within the total T cell population during malaria may result from rapid influx of malaria-specific T cells in the liver. Conversely, the absolute counts showed that the 2W1S population was actually increasing in number. While the reason for the increase in absolute number of 2W1S cells in the liver is unclear, the local inflammatory response to the parasite in this location may result in local cytokine production (IL-2, IL-7, IL-15) that drives a small amount of expansion.

We also characterized the responsiveness of this population in *P*. *yoelii-*infected or control mice by *in vivo* restimulation with heat-killed *S*. Typhimurium BRD509::2W1S. In agreement with the *in vitro* analysis, a marked decrease in the proportion of IFN-γ-producing cells was detected among CD44+ CD4 T cells, representing all CD4 T cells that have encountered antigen, as well as in the tetramer-positive 2W1S-specific CD4 T cell population (**Fig 3B and 3C**). In order to determine whether this reduction was specific to secondary stimulation with *S*. Typhimurium vaccine antigens, purified CD4 T cells from either BRD509::2W1S-vaccinated or BRD509::2W1S-vaccinated and *P*. *yoelii-*infected mice were stimulated *ex vivo* for 4 hours with the Protein Kinase C activator 12-O-Tetradecanoylphorbol-13-acetate (PMA) and the calcium ionophore ionomycin (iono). Again, we observed a decreased proportion of IFN-γ-producing cells for both the total pool of antigen-experienced T cells (CD44+) **(Fig 3D)** and among all CD4 T cells **(Fig 3E).** Together, these data suggest that active malaria parasite infection reduces the ability of memory CD4 T cells to produce IFN-γ in response to both stimulation via the T cell receptor (TCR) and to non-specific stimulation of TCR signaling pathways by PMA/iono treatment.

### Alteration in memory CD4 T cell phenotypes during malaria

**Fig 4:**
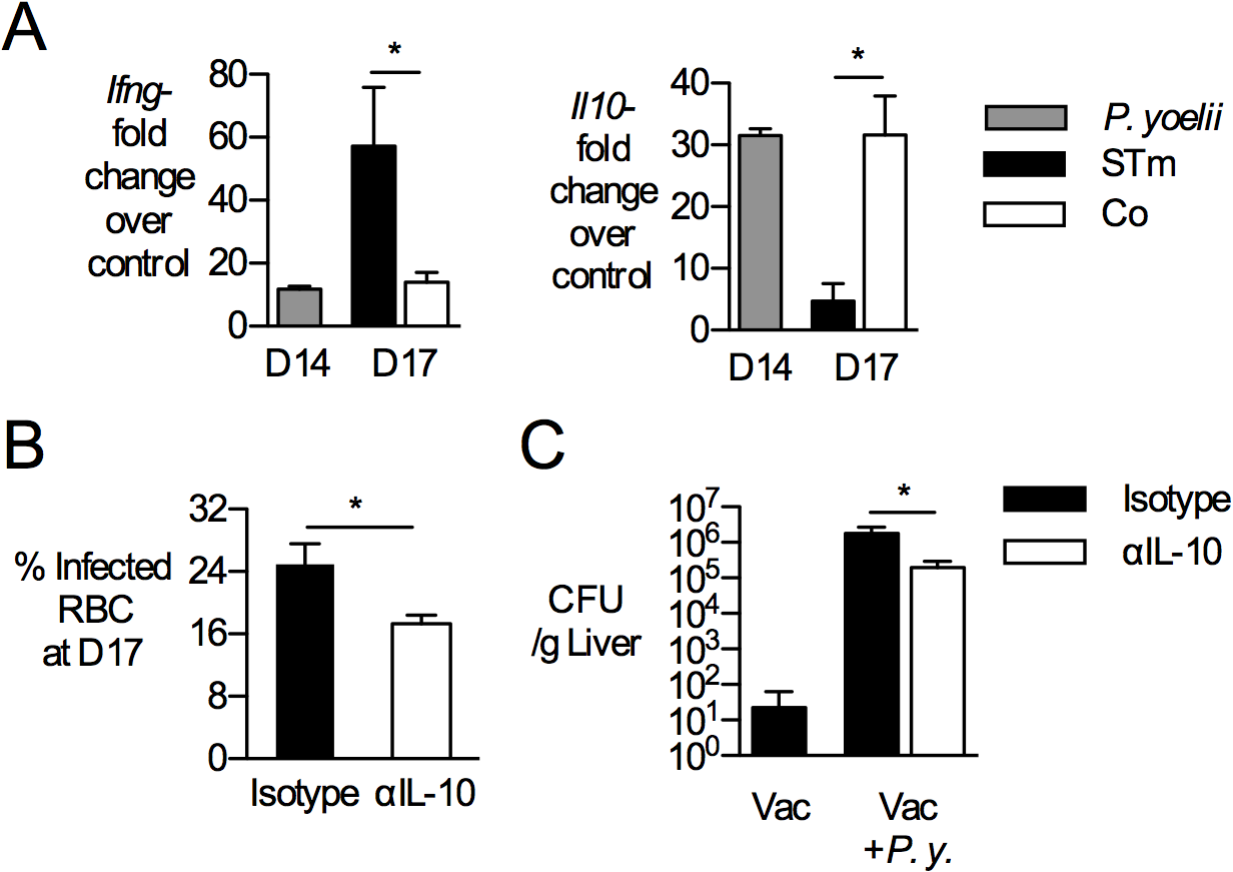
*P*. *yoelii* infection increases IL-10 expression and IL-10 blockade partially restores protection. C57BL/6 mice were vaccinated with *S*. Typhimurium BRD509::2W1S (Vac) and then given *P*. *yoelii* (*P*.*y*.) as described above. (**A**) Expression analysis of inflammatory markers by qRT-PCR at 14 d post malaria and 3 days post SL1344 challenge (D17). Transcript levels of interferon-γ (*Ifng*) and interleukin-10 (*Il10*) were determined in whole spleen tissue, normalized to beta-actin levels and shown as fold-change over mock-treated mice. Bars indicate mean+SEM (n = 4). (**B-C**) IL-10 blockade was performed as described in methods. (**B**) Parasite burden, shown as % infected red blood cells (RBCs), was determined on Giemsa-stained blood smears from tails. (**C**). Antibody-treated mice were challenged with 750 CFU of virulent *S*. Typhimurium via the i.v. route at 14 days after *P*. *yoelii* infection. At 3 d post-challenge, bacterial burden was determined in livers and normalized to tissue weight. Data represent Mean+SEM (n = 4–5 per group). Significance of differences between groups was determined using a Student’s *t* test (*, *p*<0.05; ns, not significant).

Our previous work showed that in the context of primary *S*. Typhimurium infection, IL-10 produced by phagocytes during *P*. *yoelii* infection compromised the ability of co-infected mice to control disseminated *S*. Typhimurium infection [35]. We therefore determined whether IL-10 blockade would restore protection by the NTS vaccine. Mice were vaccinated and inoculated with *P*. *yoelii* as described above, then challenged with *S*. Typhimurium in the presence or absence of neutralizing antibodies to IL-10 (**Fig 4**). As described previously, *S*. Typhimurium challenge in the context of acute *P*. *yoelii* infection resulted in dampening of IFN-γ and increase of IL-10 in the spleen of infected mice (**Fig 4A**). While IL-10 blockade reduced circulating parasite burden (**Fig 4B**), and decreased the burden of *S*. Typhimurium in the liver by one order of magnitude (**Fig 4C**), it did not restore the ability of CD4 T cells to produce IFN-γ in response to *S*. Typhimurium infection (data not shown).

**Fig 5:**
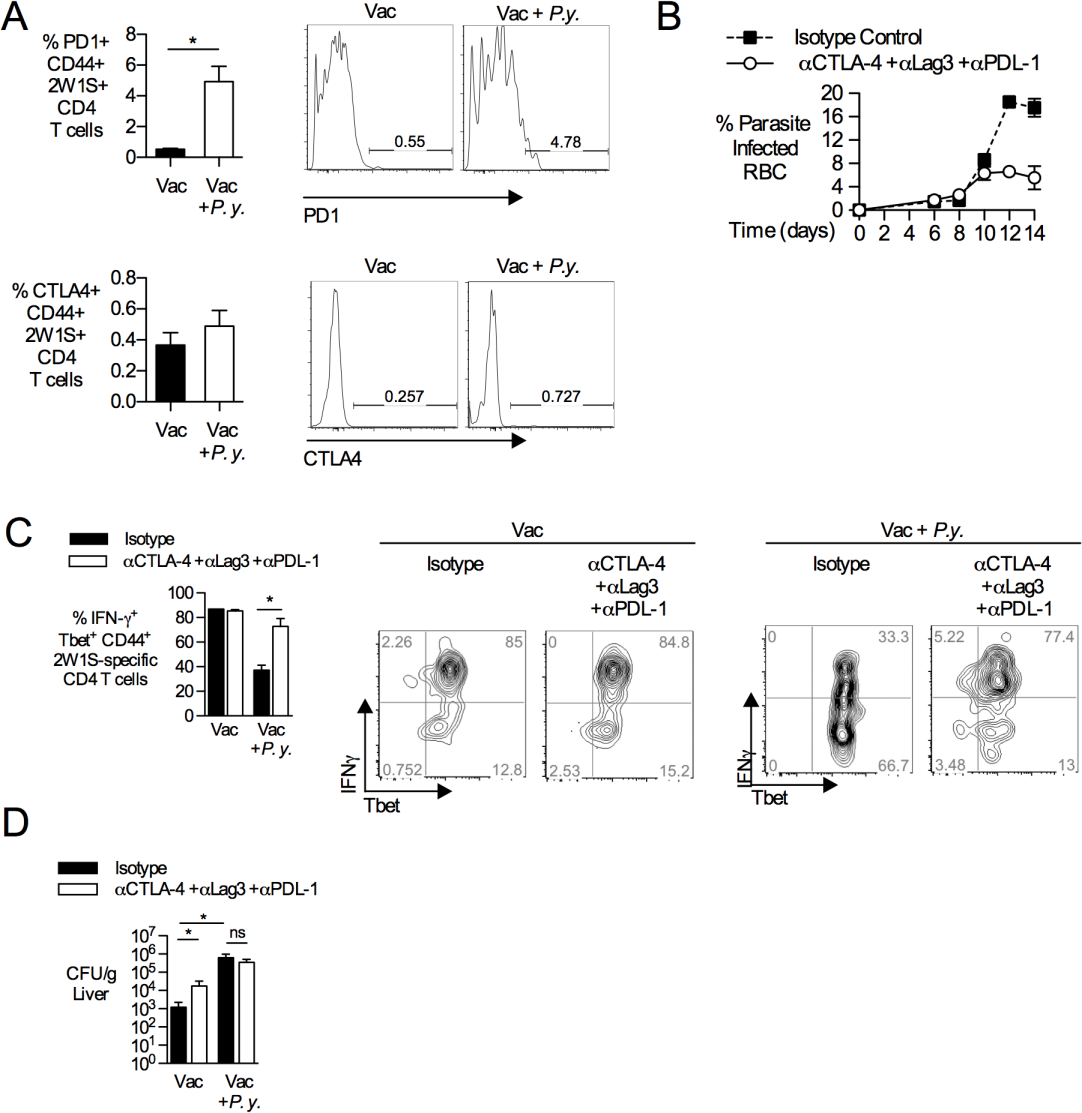
*P*. *yoelii* infection increases PD1 expression on NTS-specific CD4 T cells. C57BL/6 mice were vaccinated with *S*. Typhimurium BRD509:2W1S (Vac) and then given *P*. *yoelii* (*P*.*y*.) as described above. (**A**) At 14 d post *P*. *yoelii* infection, splenic T cells were evaluated by flow cytometry for expression of CTLA-4 and PD1. Bar graphs show percentage of CTLA-4+, and PD1+ among 2W1S-specific CD4 T cells. Data are shown as Mean+SEM (n = 10). (**B-D**) *P*. *yoelii*-infected mice were given either an antibody cocktail consisting of αCTLA-4, αLAG3 and αPDL-1 or isotype control antibodies over 7 days as described in methods. (**B**) Parasite burden with blocking antibody cocktail (n = 3) or isotype control (n = 2), as determined by blood smears. (**C**) At 14 days post-*P*. *yoelii* infection, all the treated mice were injected i.v. with heat-killed *S*. Typhimurium lysates for 4 hours. Splenic CD4 T cells were analyzed by flow cytometry for IFN-γ production. Bar graphs show percentage of IFN-γ producing Tbet+ CD44+ 2W1S-specific CD4 T cells with blocking antibody cocktail (n = 3) or isotype control (n = 2). (**D**) Antibody-treated mice were challenged with 750 CFU of virulent *S*. Typhimurium via the i.v. route at 14 days after *P*. *yoelii* infection. At 3 d post-challenge, bacterial burden was determined in livers and normalized to tissue weight. Data represent Mean+SEM (n = 5 per group). Data are shown as Mean+SEM. Significance of differences between groups was determined using a Student’s *t* test (*, *p*<0.05; ns, not significant).

Memory T cells can express inhibitory molecules that regulate effector function upon activation with cognate antigen [36]. Increased expression of inhibitory molecules, especially PD-1, CTLA4 and LAG3, has been associated with T cell exhaustion, a state of reduced effector function that arises during chronic infection [37]. Therefore, we determined the effect of acute malaria parasite infection on expression of inhibitory receptors by antigen-specific CD4 T cells in *S*. Typhimurium-vaccinated mice. At the peak of malaria infection, CD44+ 2W1S-tetramer-positive CD4 T cells demonstrated increased surface expression of PD-1, but not CTLA4 (**Fig 5A).** Since CD4 T cells are known to express multiple inhibitory molecules during infection with murine malaria parasites [38,39], we determined whether expression of inhibitory molecules was responsible for limiting the effector function of 2W1S-specific T cells after *S*. Typhimurium vaccination. To this end, we examined the effect of antibody blockade of CTLA-4, LAG3 and the PD-1 ligand PD-L1. As previously described [38,39], antibody blockade diminished circulating parasite levels **(Fig 5B)**. Following *in vivo* stimulation with heat-killed *S*. Typhimurium, simultaneous blockade of CTLA-4, LAG3 and PD-L1 increased IFN-γ production from 2W1S-specific CD4 T cells (**Fig 5C**). This result suggested that expression of inhibitory molecules could contribute to the observed erosion of vaccine-induced T cell memory during malaria. However, blockade of CTLA-4, LAG3 and PD-L1 was insufficient to restore control of *S*. Typhimurium infection (**Fig 5D**), suggesting that additional effects of malaria on immunity to *S*. Typhimurium may be responsible for loss of vaccine-mediated protection. Together, our results suggest that multiple effects of malaria parasite infection converge to compromise immunity to NTS induced by a live, attenuated vaccine.

## Discussion

Clinical studies suggest that natural adaptive immunity to NTS is widespread among children in Sub-Saharan Africa [40]. Here we show that during malaria infection, adaptive immunity to NTS infection conferred by previous exposure is temporarily interrupted. In mouse infection models, adaptive immunity to *Salmonella* serovars is conferred by both antibody and T cells [27,40,41]. Malaria can cause apoptosis of memory B cells and long-lived plasma cells in mice [42–44], suggesting a potential effect on antibody responses. However, during the time frame of our study, which encompassed active and resolving malaria, circulating antibody levels induced by *S*. Typhimurium vaccination were maintained. Therefore, our studies with a live, attenuated vaccine do not permit any conclusions on the effect of malaria on immunity conferred by glycoconjugate vaccines, which elicit antibody-mediated immunity [12]. We observed that the loss in vaccine-induced protection in *P*. *yoelii*-infected mice correlated with increased IL-10 expression, as well as with the suppression of CD4 and CD8 T cell effector responses that are specific for *S*. Typhimurium antigens.

In contrast to what has been shown for B cells, memory CD4 T cells did not appear to undergo apoptosis during malaria, since absolute numbers of vaccine-induced, antigen-specific CD4 T cells were maintained at the peak of malaria parasite infection (**Fig 3A**). Nevertheless, antigen-specific memory CD4 T cells failed to respond to antigen by differentiating into IFN-γ- producing effector cells. The refractory state of memory CD4 T cells did not result solely from previously described effects of malaria on DC function [45], as antigen-presenting cells isolated from *P*. *yoelii*-infected mice were able to prime vaccine-elicited memory CD4 T cells specific for the 2W1S epitope (data not shown). Furthermore, memory CD4 T cells from *P*. *yoelii*-infected mice were unable to respond *ex vivo* to *S*. Typhimurium Class II antigens flagellin, SseI and SseJ presented by naïve splenocytes, providing further evidence for a T cell-intrinsic defect in adaptive immunity during malaria (**Fig 2**). This intrinsic defect in IFN-γ-production was observed with *in vivo* antigen challenge as well as *ex vivo* stimulation with PMA/iono (**Fig 3B–3E**). With increased surface expression of PD1 on memory CD4 T cells (**Fig 5A**), the phenotypic traits of *Salmonella* vaccine-induced memory T cells during malaria share characteristics of exhausted CD4 T cells identified during chronic viral infection [46]. This finding is consistent with previous reports of CD4 T cell dysfunction during malaria [38,47], which has been shown to have the beneficial effect of preventing immunopathology during acute infection [48,49]. Blockade of PDL-1, CTLA4 and LAG3 restored the ability of antigen-specific CD4 T cells to produce IFN-γ, suggesting that in addition to its previously described effect on control of acute malaria [38], upregulation of inhibitory ligands contributes to dysfunction of memory CD4 T cells generated by vaccination. However, this effect alone is insufficient to explain loss of vaccine-induced immunity (**Fig 5D**). Since IL-10 blockade partially restored protection against NTS in the context of concurrent *P*. *yoelii* infection (**Fig 4C**), it is likely that the suppressive effect of IL-10 on macrophage bactericidal function is a contributing factor to loss of immunity, similar to what we have reported previously in the context of primary *S*. Typhimurium infection [35]. It is interesting to note in this context that both IL-10 blockade and blockade of PDL-1, CTLA4 and LAG3 reduced the level of circulating malaria parasites, however only IL-10 blockade restored vaccine-mediated protection. Thus, while reduction of parasitemia may have contributed to the improved control of *S*. Typhimurium after IL-10 blockade, it may not be sufficient on its own to restore immune responses during the acute phase of infection.

During acute malaria, it is likely that a defect in memory CD4 T cell effector function acts together with other malaria-induced defects in innate immunity to compromise established adaptive immunity acquired either by vaccination or from previous infections. These include reduced MHCII expression [45,50,51], altered macrophage polarization [35], neutrophil dysfunction [52] and depletion of complement [53]. Further, in agreement with our results (Fig 2) a loss of memory CD8 T cells induced by both bacterial and viral infections was observed in a co-infection model [54]. Given these numerous effects of malaria on innate and adaptive immunity, it is possible that interventions to restore adaptive immune function may fail to compensate for the effects of malaria parasite infection on macrophages and neutrophils that are critical to control of NTS infection.

Some limitations of our study design should be taken into consideration when extrapolating our results to vaccination of children with malaria. We do not know the mechanism by which malaria parasite infection suppressed memory T cell responses in our animal model, and additional studies will be required to define the precise defects. Further, in order to assay antigen-specific Th1 cell responses, we utilized parenteral routes of infection and some of our assays used stimulation of T cells with antigen *ex vivo* under conditions that may not precisely mimic those encountered in the host environment. In addition, our murine malaria model utilizing *P*. *yoelii* infection has some differences to *P*. *falciparum* malaria that is widespread in Africa, including higher levels of circulating parasites, and there may be differences in responses of mice and humans to malaria parasite infection. Also, since live attenuated vaccines, but not polysaccharide conjugate vaccines depend on cellular immunity, the loss of T cell-based immunity would not be expected to affect responses to glycoconjugate vaccines. For these reasons, clinical studies would be needed to assess whether loss of cellular immune responses to *S*. Typhimurium occurs in children with malaria and whether this loss of T cell immunity extends to vaccines against other intracellular pathogens, which would be suggestive of global suppression of T cell function by malaria.

Taken together, our results with an animal model suggest that multiple effects of malaria on the immune system can compromise established adaptive immunity to a live, attenuated *S*. Typhimurium vaccine during the acute phase of malaria. If a similar loss of adaptive immune responses is found to occur in children with malaria, it would suggest that malaria eradication efforts may have the added benefit of increasing vaccine-mediated protection against disseminated non-typhoidal *Salmonella* infection.

## Supporting Information

S1 Fig Effect of *P*. *yoelii* infection on spleen size, anemia and circulating lymphocytes. Relating to Fig 1, C57BL/6 mice were infected with 4x10^7^ infected red blood cells (RBC) of *P*. *yoelii* by the i.p. route, and the rest given control blood. **(A)** Picture and weights (n = 4) of spleens taken from *P*. *yoelii*-infected or control mice at 14 and 28 days post-infection. **(B)** Bar graphs represent data from complete blood counts taken from blood obtained through cardiac puncture at either 14 or 28 days post malaria parasite infection (n = 4). **(C)** Sub-patent malaria infection was determined by transfer of 0.2mL of whole blood from mice at 28 days post-*P*. *yoelii* infection into naïve mice (n = 4). Parasite burden was monitored by blood smears at 2, 5, and 12 days post transfer but no parasites were detected (ND, not detected). Data represents Mean+SEM. Significance of differences between groups was determined using a Student’s *t* test (*, *p*<0.05; ns, not significant).
(TIFF)

S2 Fig Effect of malaria parasite infection on systemic bacterial burden in *S*. Typhimurium-vaccinated mice after challenge with virulent *S*. Typhimurium. Relating to Fig 1, C57BL/6 mice were vaccinated i.v. with 5x10^5^ attenuated *S*. Typhimurium BRD509. 42 days later, a group of vaccinated mice was infected with 4x10^7^ infected red blood cells (RBC) containing *P*. *yoelii* by the i.p. route, and the rest given control blood. Bacterial burden in the spleens of mice challenged with virulent *S*. Typhimurium at either 14 days (A) or 28 days (B) post *P*. *yoelii* infection (n = 4–8). (C) C57BL/6 (n = 5), which are *Nramp1*/*Slc11a1* ^*-/-*^, or C57BL/6 expressing a functional allele of *Slc11a1* (n = 3–13), were vaccinated, then inoculated with *P*. *yoelii* and challenged i.v. with virulent *S*. Typhimurium at 14 days after *P*. *yoelii* infection. *S*. Typhimurium burden was quantified in the spleen (top) and blood (bottom) at 3 days after challenge with virulent *S*. Typhimurium. ND, not detected. X, not performed. Data are shown as Mean+SEM. Significance of differences between groups was determined using a Student’s *t* test (*, *p*<0.05; ns, not significant).
(TIFF)

S3 Fig Systemic bacterial burden in CBA/J mice vaccinated against *S*. Typhimurium, infected with malaria and then challenged intragastrically with virulent *S*. Typhimurium. Relating to Fig 1, CBA/J mice were vaccinated i.v. with 5x10^5^ CFU of attenuated *S*. Typhimurium BRD509. 42 days later, a group of vaccinated mice was infected with 4x10^7^ infected red blood cells (RBC) of the non-lethal murine malaria parasite *P*. *yoelii* by the i.p. route, and the rest given control blood. (A) Parasite burden, shown as % infected red blood cells (RBC), was determined from Giemsa-stained blood smears (n = 4). (B) Circulating red blood cells at 14 days post-*P*. *yoelii* infection in CBA/J mice was determined by manual counting (n = 7). (C) Bacterial burden in the liver (left panel) and spleen (right panel) was determined 4 days after i.g. challenge with 1x10^8^ CFU of virulent *S*. Typhimurium (n = 6–7). Data are shown as Mean+SEM. Significance of differences between groups was determined using a Student’s *t* test (*, *p*<0.05; ns, not significant).
(TIFF)

S4 Fig Circulating IgG2c and IgM to *Salmonella* remains during malaria-parasite infection. C57BL/6 mice were vaccinated with *Salmonella* BRD509 and on day 42, these mice were inoculated with *P*. *yoelii* as described previously. At either 14 days (top) or 28 days (bottom) post-*P*. *yoelii* infection,levels of circulating *Salmonella*-specific IgG2c (A) or IgM (B) was determined by antibody ELISA through serial dilution of serum bound to plates coated with heat-killed *Salmonella*. Data are shown as Mean±SEM (n = 3–4).
(TIFF)

S5 Fig Similar numbers of IFN-γ producing, *Salmonella*-specific CD4 T cells recovered from lymph nodes. Relating to Fig 3, C57BL/6 mice were vaccinated i.v. with 5x10^5^ CFU attenuated *S*. Typhimurium BRD509-2W1S. 42 days later, groups of vaccinated mice were inoculated i.p. with either 4x10^7^ *P*. *yoelii*-infected RBC or with control blood. **(A)** Representative dot plots relating to graphs in Fig 3A. Plots are 2W1S-CD4 T cells from the liver and spleen shown as a percent of total CD4 T cells. **(B)** CD4 T cells from mesenteric lymph nodes (MesLN) and peripheral lymph nodes (pLNs) were analyzed by flow cytometry. Using 2W1S::I-A^b^ tetramer staining on CD4 T cells, we evaluated both the percentage (%) and absolute number (#) of 2W1S-specific CD4 T cell population in MesLN and pLNs. Bar represents Mean+SEM (n = 3–4). Significance of differences between groups was determined using a Student’s *t* test (*, *p*<0.05; ns, not significant).
(TIFF)
