# Supplementary figures and images for: Transient Loss of Protection Afforded by a Live Attenuated Non-typhoidal Salmonella Vaccine in Mice Co-infected with Malaria
Source: PLoS Negl Trop Dis. 2015 Sep 14;9(9):e0004027. doi: 10.1371/journal.pntd.0004027 (PMC4569369; doi:10.1371/journal.pntd.0004027)

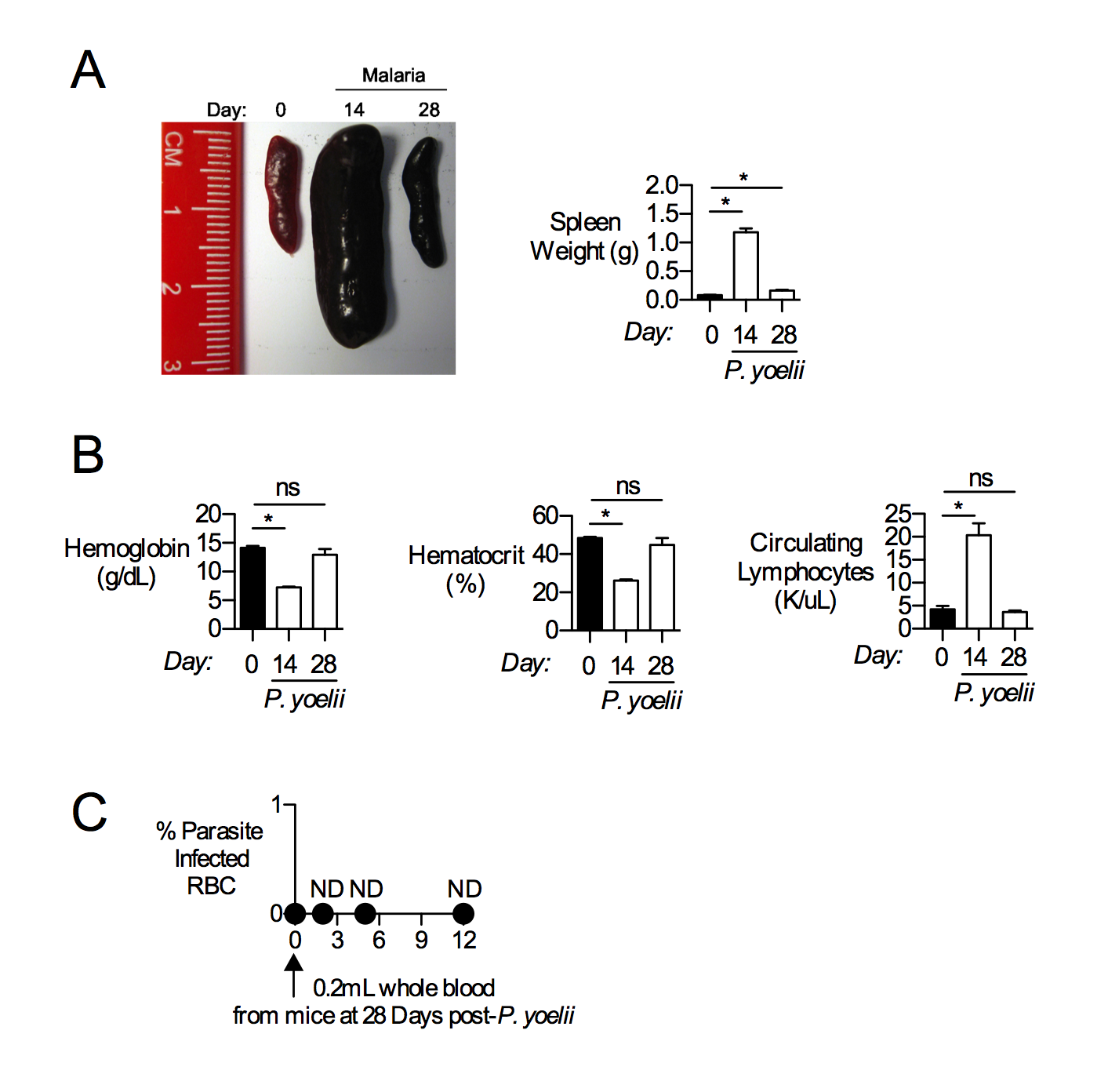

Supplement: S1 Fig — Relating to Fig 1, C57BL/6 mice were infected with 4x10^7^ infected red blood cells (RBC) of P. yoelii by the i.p. route, and the rest given control blood. (A) Picture and weights (n = 4) of spleens taken from P. yoelii-infected or control mice at 14 and 28 days post-infection. (B) Bar graphs represent data from complete blood counts taken from blood obtained through cardiac puncture at either 14 or 28 days post malaria parasite infection (n = 4). (C) Sub-patent malaria infection was determined by transfer of 0.2mL of whole blood from mice at 28 days post-P. yoelii infection into naïve mice (n = 4). Parasite burden was monitored by blood smears at 2, 5, and 12 days post transfer but no parasites were detected (ND, not detected). Data represents Mean+SEM. Significance of differences between groups was determined using a Student’s t test (*, p<0.05; ns, not significant). (TIFF) [file pntd.0004027.s001.tiff]

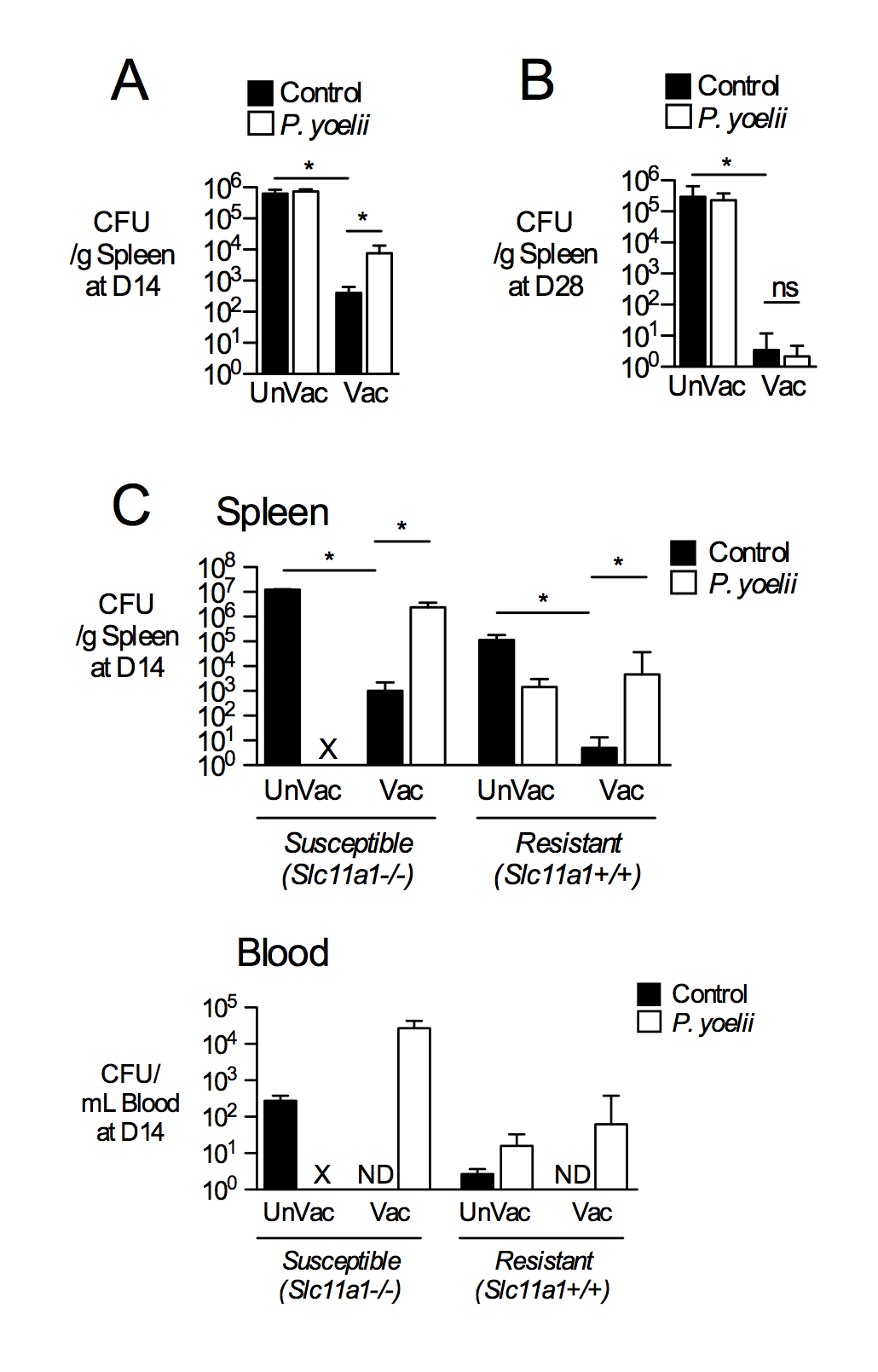

Supplement: S2 Fig — Relating to Fig 1, C57BL/6 mice were vaccinated i.v. with 5x10^5^ attenuated S. Typhimurium BRD509. 42 days later, a group of vaccinated mice was infected with 4x10^7^ infected red blood cells (RBC) containing P. yoelii by the i.p. route, and the rest given control blood. Bacterial burden in the spleens of mice challenged with virulent S. Typhimurium at either 14 days (A) or 28 days (B) post P. yoelii infection (n = 4–8). (C) C57BL/6 (n = 5), which are Nramp1/Slc11a1 -/-, or C57BL/6 expressing a functional allele of Slc11a1 (n = 3–13), were vaccinated, then inoculated with P. yoelii and challenged i.v. with virulent S. Typhimurium at 14 days after P. yoelii infection. S. Typhimurium burden was quantified in the spleen (top) and blood (bottom) at 3 days after challenge with virulent S. Typhimurium. ND, not detected. X, not performed. Data are shown as Mean+SEM. Significance of differences between groups was determined using a Student’s t test (*, p<0.05; ns, not significant). (TIFF) [file pntd.0004027.s002.tiff]

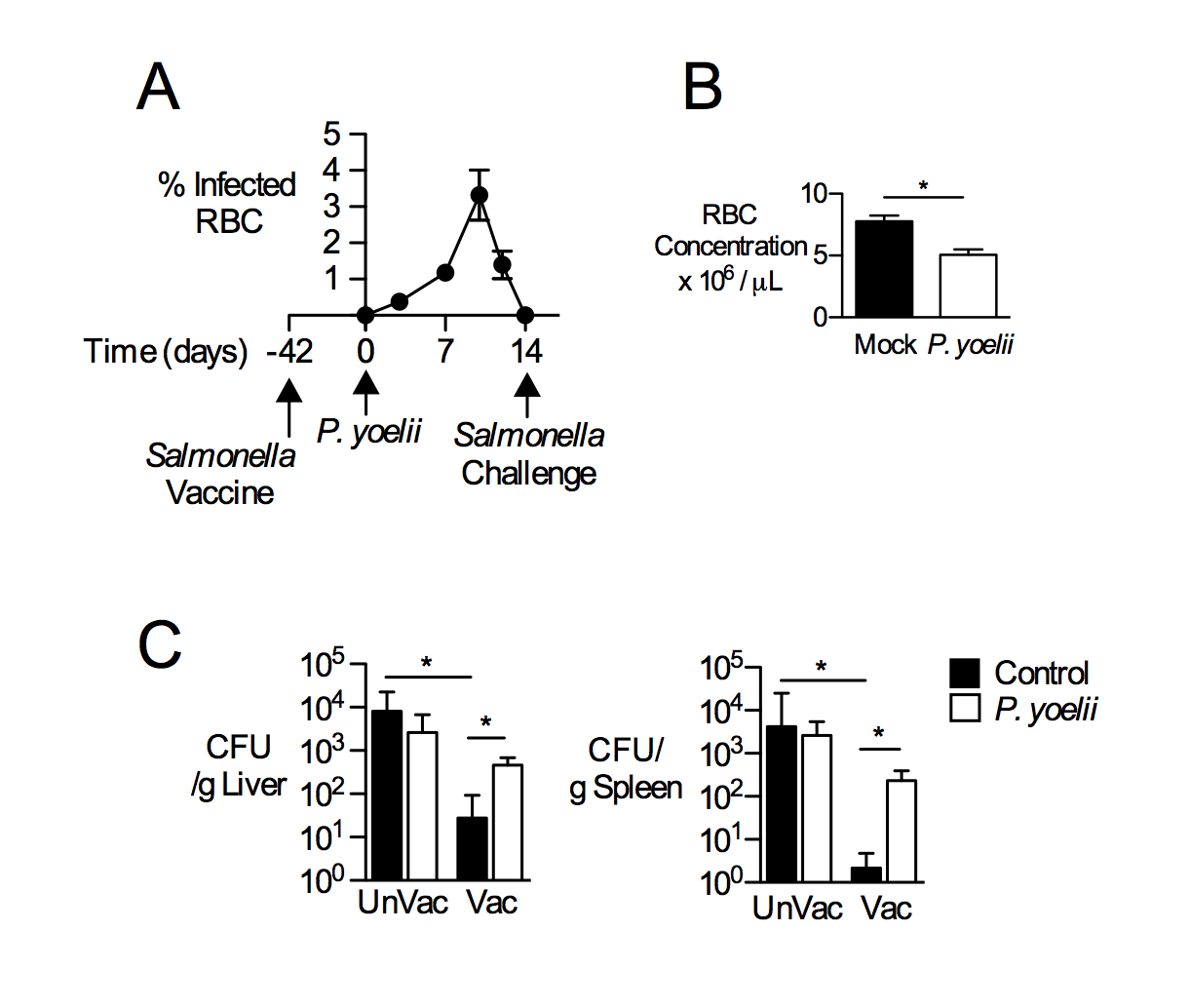

Supplement: S3 Fig — Relating to Fig 1, CBA/J mice were vaccinated i.v. with 5x10^5^ CFU of attenuated S. Typhimurium BRD509. 42 days later, a group of vaccinated mice was infected with 4x10^7^ infected red blood cells (RBC) of the non-lethal murine malaria parasite P. yoelii by the i.p. route, and the rest given control blood. (A) Parasite burden, shown as % infected red blood cells (RBC), was determined from Giemsa-stained blood smears (n = 4). (B) Circulating red blood cells at 14 days post-P. yoelii infection in CBA/J mice was determined by manual counting (n = 7). (C) Bacterial burden in the liver (left panel) and spleen (right panel) was determined 4 days after i.g. challenge with 1x10^8^ CFU of virulent S. Typhimurium (n = 6–7). Data are shown as Mean+SEM. Significance of differences between groups was determined using a Student’s t test (*, p<0.05; ns, not significant). (TIFF) [file pntd.0004027.s003.tiff]

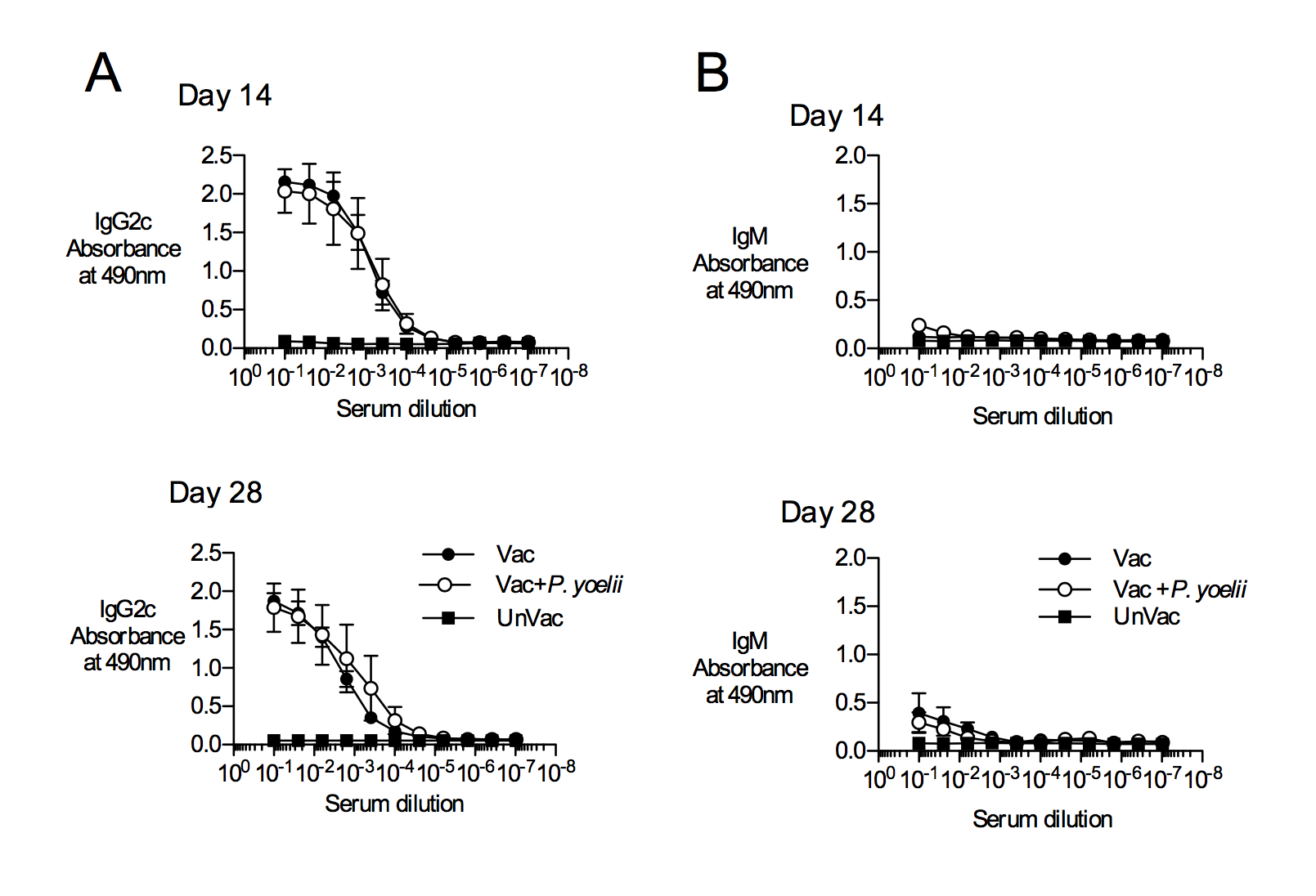

Supplement: S4 Fig — C57BL/6 mice were vaccinated with Salmonella BRD509 and on day 42, these mice were inoculated with P. yoelii as described previously. At either 14 days (top) or 28 days (bottom) post-P. yoelii infection,levels of circulating Salmonella-specific IgG2c (A) or IgM (B) was determined by antibody ELISA through serial dilution of serum bound to plates coated with heat-killed Salmonella. Data are shown as Mean±SEM (n = 3–4). (TIFF) [file pntd.0004027.s004.tiff]

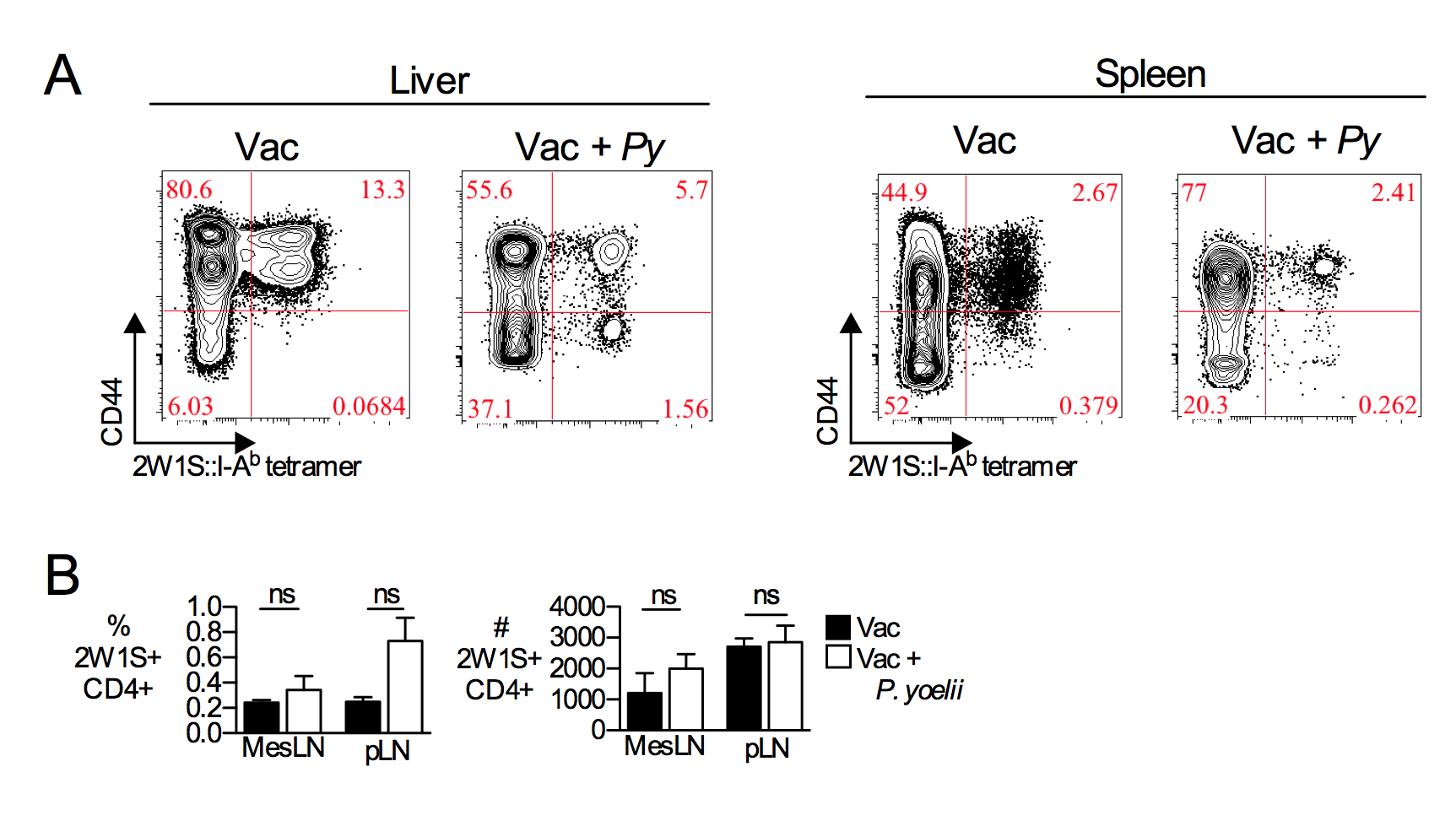

Supplement: S5 Fig — Relating to Fig 3, C57BL/6 mice were vaccinated i.v. with 5x10^5^ CFU attenuated S. Typhimurium BRD509-2W1S. 42 days later, groups of vaccinated mice were inoculated i.p. with either 4x10^7^ P. yoelii-infected RBC or with control blood. (A) Representative dot plots relating to graphs in Fig 3A. Plots are 2W1S-CD4 T cells from the liver and spleen shown as a percent of total CD4 T cells. (B) CD4 T cells from mesenteric lymph nodes (MesLN) and peripheral lymph nodes (pLNs) were analyzed by flow cytometry. Using 2W1S::I-Ab tetramer staining on CD4 T cells, we evaluated both the percentage (%) and absolute number (#) of 2W1S-specific CD4 T cell population in MesLN and pLNs. Bar represents Mean+SEM (n = 3–4). Significance of differences between groups was determined using a Student’s t test (*, p<0.05; ns, not significant). (TIFF) [file pntd.0004027.s005.tiff]
